# Compressed graph representation for scalable molecular graph generation

**DOI:** 10.1186/s13321-020-00463-2

**Published:** 2020-09-23

**Authors:** Youngchun Kwon, Dongseon Lee, Youn-Suk Choi, Kyoham Shin, Seokho Kang

**Affiliations:** 1 Samsung Advanced Institute of Technology, Samsung Electronics Co. Ltd., 130 Samsung-ro, Yeongtong-gu, Suwon, Republic of Korea; 2 Department of Computer Science and Engineering, Seoul National University, 1 Gwanak-ro, Gwanak-gu, Seoul, Republic of Korea; 3 Department of Industrial Engineering, Sungkyunkwan University, 2066 Seobu-ro, Jangan-gu, Suwon, Republic of Korea

**Keywords:** Molecular graph generation, Compressed graph representation, Graph variational autoencoder, Deep learning

## Abstract

Recently, deep learning has been successfully applied to molecular graph generation. Nevertheless, mitigating the computational complexity, which increases with the number of nodes in a graph, has been a major challenge. This has hindered the application of deep learning-based molecular graph generation to large molecules with many heavy atoms. In this study, we present a molecular graph compression method to alleviate the complexity while maintaining the capability of generating chemically valid and diverse molecular graphs. We designate six small substructural patterns that are prevalent between two atoms in real-world molecules. These relevant substructures in a molecular graph are then converted to edges by regarding them as additional edge features along with the bond types. This reduces the number of nodes significantly without any information loss. Consequently, a generative model can be constructed in a more efficient and scalable manner with large molecules on a compressed graph representation. We demonstrate the effectiveness of the proposed method for molecules with up to 88 heavy atoms using the GuacaMol benchmark.

## Introduction

Deep learning has revolutionized the design of novel molecules required for real-world industrial applications. Whereas traditional approaches have mostly been based on human knowledge and intuition, the use of deep learning has enabled the autonomous design of molecules by learning from previously accumulated data [1–3]. Most existing methods use deep generative models, such as variational autoencoders (VAEs) and generative adversarial networks (GANs). Their capabilities depend on the way of representing a molecule. Such representations include simplified molecular-input line-entry system (SMILES) and molecular graph representation. Although the SMILES representation has been demonstrated to be useful, recent research tends to employ the molecular graph representation, which is a natural and intuitive way of representing a molecule by regarding its atoms and bonds as nodes and edges, respectively [1].

A major challenge for molecular graph generation is addressing the scalability issue caused by its high computational complexity [4]. The representation of a molecular graph $\mathcal {G}=(\mathcal {V},\mathcal {E})$ on which a model learns, where $\mathcal {V}$ and $\mathcal {E}$ are the set of nodes and edges in $\mathcal {G}$, typically involves an adjacency expression between its nodes, yielding $\mathcal {O}(|\mathcal {V}|^2)$ complexity. A naïve approach is to regard only heavy atoms in a molecule as nodes in the corresponding graph representation by treating hydrogen atoms implicitly as node features. This approach is however not scalable for large molecules with many heavy atoms, which are abundant in the real world [5, 6]. Consequently, existing methods were evaluated by limiting the size of the molecules in the training dataset, which was often set to less than 50 heavy atoms. The benchmark datasets with small molecules, such as QM9 [7, 8] and ZINC [9], have been commonly employed in the literature.

For scalable molecular graph generation, there have been research attempts to alleviate the complexity $\mathcal {O}(|\mathcal {V}|^2)$ via representational simplification. One approach involves representing a molecular graph as a sequence of vectors and then building an autoregressive model on the sequence representation for the sequential generation of nodes and edges that form a graph. You et al. presented GraphRNN which constructs a model on a node-level sequence representation with *M*-dimensional adjacency vectors, where *M* is set to less than $|\mathcal {V}|$, by employing breadth-first-search node ordering with which the complexity is reduced to $\mathcal {O}(|\mathcal {V}|M)$ [10]. Goyal et al. presented GraphGen which transforms a molecular graph into an edge-level sequence based on minimum depth-first-search coding, which leads to a complexity of $\mathcal {O}(|\mathcal {E}|)$ [4]. However, as in the SMILES representation, the sequential nature imposes constraints on the model architecture and prevents the model from capturing molecular similarity and retaining chemical validity. Another approach is to reduce the number of nodes $|\mathcal {V}|$ directly in the representation. Jin et al. presented junction tree VAE (JTVAE) which represents a molecular graph as a junction tree, whose nodes correspond to valid chemical substructures, using tree decomposition [11]. The compressed representation can be generally applicable to any model architecture. Nevertheless, JTVAE can suffer from high dimensionality due to the dramatic increase in the number of node features, because of the large variety of chemical substructures that appear in the dataset.

For a more practical application of molecular graph generation, we focus on the latter approach which involves reducing the number of nodes directly in the representation. This study aims to improve the scalability of molecular graph generation to large molecules while maintaining the capability of generating chemically valid and diverse molecular graphs. We present a novel method for the compression of molecular graph representation for scalable molecular graph generation. We designate six small substructural patterns that commonly appear between two heavy atoms in practice and regard their appearances as additional edge features along with the bond types. A molecular graph is compressed by substituting the relevant substructures with new edges. This compression reduces the number of nodes without drastically increasing the number of edge features, making it scalable to large molecules. In addition, the compressed graph can be reconstructed into the original graph without any information loss.

## Methods

### Molecular graph compression

The conventional graph representation of a molecule is an undirected graph whose nodes and edges correspond to heavy atoms and their bonds in the molecule, respectively. Hydrogen atoms are treated implicitly as node features, and thus, they are not regarded as explicit nodes. Formally, a molecular graph is defined as $\mathcal {G}=(\mathcal {V},\mathcal {E})$, where $\mathcal {V}$ and $\mathcal {E}$ denote the sets of nodes and edges, respectively. Each node corresponding to the *i*-th heavy atom is represented by a node vector $\mathbf {v}^i \in \mathcal {V}$ with the dimensionality of *p*, whose features indicate the atom type, formal charge, and valence information. An edge corresponding to the connection between the *i*-th and *j*-th atoms is represented by an edge vector $\mathbf {e}^{i,j} \in \mathcal {E}$ with a dimensionality of *q*, whose features are associated with a bond type. The property vector $\mathbf {y}=(y^1,\ldots ,y^{l})$ represents the properties of the molecule.

**Fig. 1:**
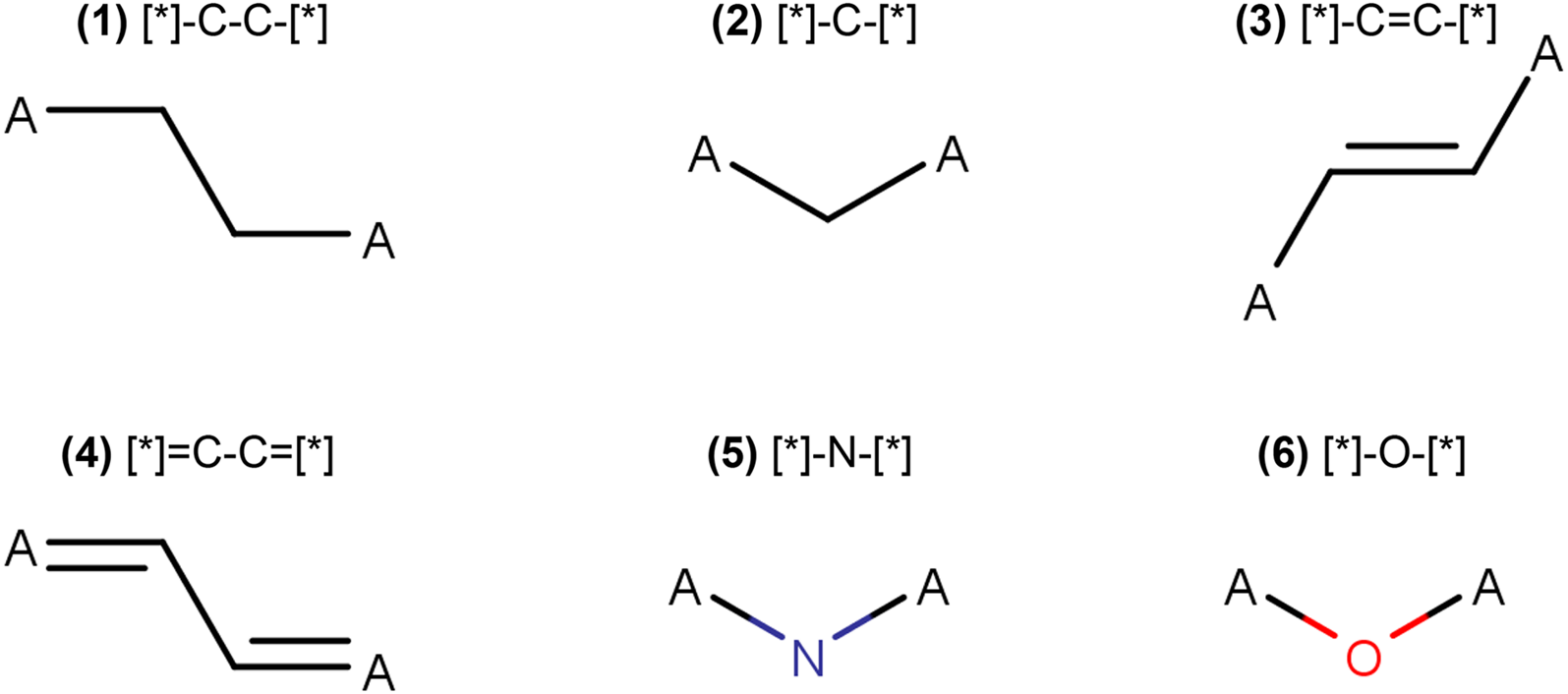
Substructural patterns that commonly appear between two atoms in molecules

We compress the graph representation by reducing the number of nodes. We employ six small substructural patterns that commonly appear between two heavy atoms, which are listed in Fig. 1. Each of the substructural patterns contains only one or two heavy atoms with the atom types corresponding to C, N, and O, which are abundant in real-world molecules. We represent the appearances of these six substructural patterns using additional edge features, which may be sufficient for most real-world datasets. Nevertheless, depending on the training dataset, we can additionally designate more substructural patterns to be regarded as edge features for further compression.

Formally, we define a compression function $\Phi$ that compresses an input graph. For an original graph $\mathcal {G}$, the corresponding compressed graph $\mathcal {G}'$ is obtained using the function $\Phi$ as

1 $$\begin{aligned} \mathcal {G}'=\Phi {(\mathcal {G})}. \end{aligned}$$

Given the input graph $\mathcal {G}$, the function $\Phi$ finds the substructures that are relevant to the six designated patterns. With canonical ordering of the atoms in $\mathcal {G}$, each substructure is sequentially converted to an edge by representing its appearance using the corresponding edge feature. The canonical numbers of atoms are used to prioritize which substructure is converted first. When multiple substructures overlap, the one with non-overlapping atoms having smaller canonical numbers is chosen to be replaced by an edge.

With the addition of edge features, the edge vector of compressed graph $\mathcal {G}'$ has higher dimensionality than that of the original graph $\mathcal {G}$. This compression reduces one or two nodes per substructure. There may exist multiple substructures in between an atom pair, and a larger molecule may contain more relevant substructures. A graph will be further compressed if more of the substructural patterns exist in it.

**Fig. 2:**
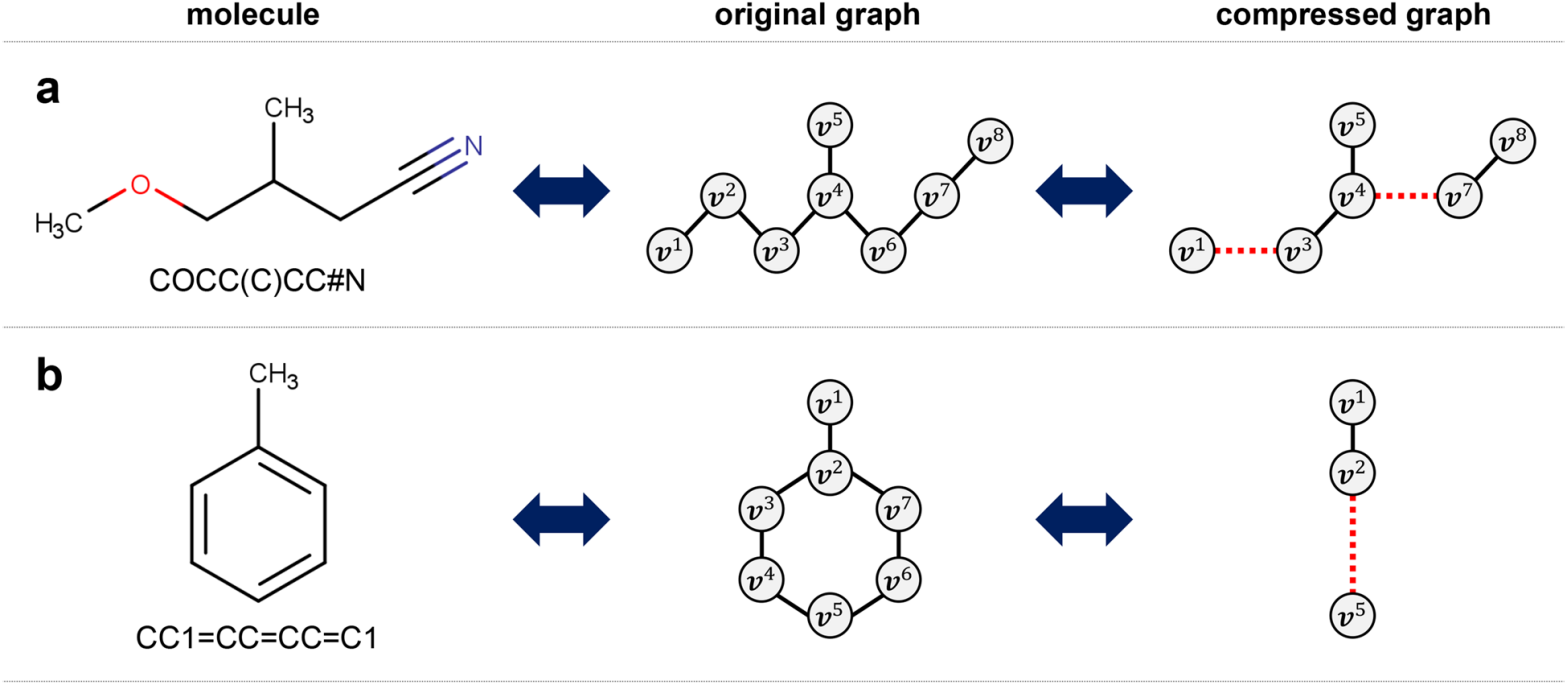
Example of compressed graph representation

Figure 2 shows an illustrative example of the compressed graph representation for two molecules. In the first example, the original graph contains eight nodes because the corresponding molecule has eight heavy atoms. For the original graph, the substructures 1-2-3, 2-3-4, and 4-6-7 are relevant to patterns 6, 2, and 2, respectively. The substructures 1-2-3 and 2-3-4 overlap, and therefore, one among them needs to be chosen for compression. Because 1-2-3 has smaller canonical numbers, we choose 1-2-3 to be replaced. After 1-2-3 and 2-3-4 are replaced by the respective edges, the number of nodes is reduced to six. The second example involves an original graph that contains seven nodes. Two substructures, 2-3-4-5 with pattern 3 and 2-7-6-5 with pattern 4, appear simultaneously between the 2nd and 5th nodes. After they are substituted by edges, the compressed graph contains three nodes.

The main advantages of compressed graph representation are as follows. Firstly, the compressed representation reduces the number of nodes (i.e., $|\mathcal {V}'| \le |\mathcal {V}|$), thereby providing better scalability to large molecules. Secondly, the compression is reversible, meaning that the compressed graph can be reconstructed into the original one without any information loss using a decompression function $\Phi ^{-1}$ (i.e., $\mathcal {G}=\Phi ^{-1}{(\Phi {(\mathcal {G})})}$). Thirdly, it does not drastically increase the dimensionality of edge vectors because only pre-chosen substructural patterns are additionally involved as edge features in the compressed representation (i.e., $q'-q$ is a small constant). The increase in edge dimensionality does not significantly affect the scalability.

### Learning on graph representation

In this study, we build a non-autoregressive graph VAE (NAGVAE), presented in [12], on the compressed graph representation. The model seeks to find the generative distribution $p_\theta (\mathcal {G}|\mathbf {z},\mathbf {y})$ parameterized by $\theta$. The prior distributions $p(\mathbf {z})$ and $p(\mathbf {y})$ are set to $\mathcal {N}(\mathbf {z}|\mathbf {0},\mathbf {I})$ and $\mathcal {N}(\mathbf {y}|\varvec{\mu }_\mathbf {y},\varvec{\Sigma }_\mathbf {y})$, respectively. We introduce an approximate posterior distribution $q_\phi (\mathbf {z}|\mathcal {G},\mathbf {y})=\mathcal {N}(\mathbf {z}|{\varvec{\mu }_\mathbf {z}(\mathcal {G},\mathbf {y}), \text {diag}(\varvec{\sigma }^2_\mathbf {z}(\mathcal {G},\mathbf {y})}))$ parameterized by $\phi$ to address the intractability of the posterior distribution $p_\theta (\mathbf {z}|\mathcal {G},\mathbf {y})$.

**Fig. 3:**
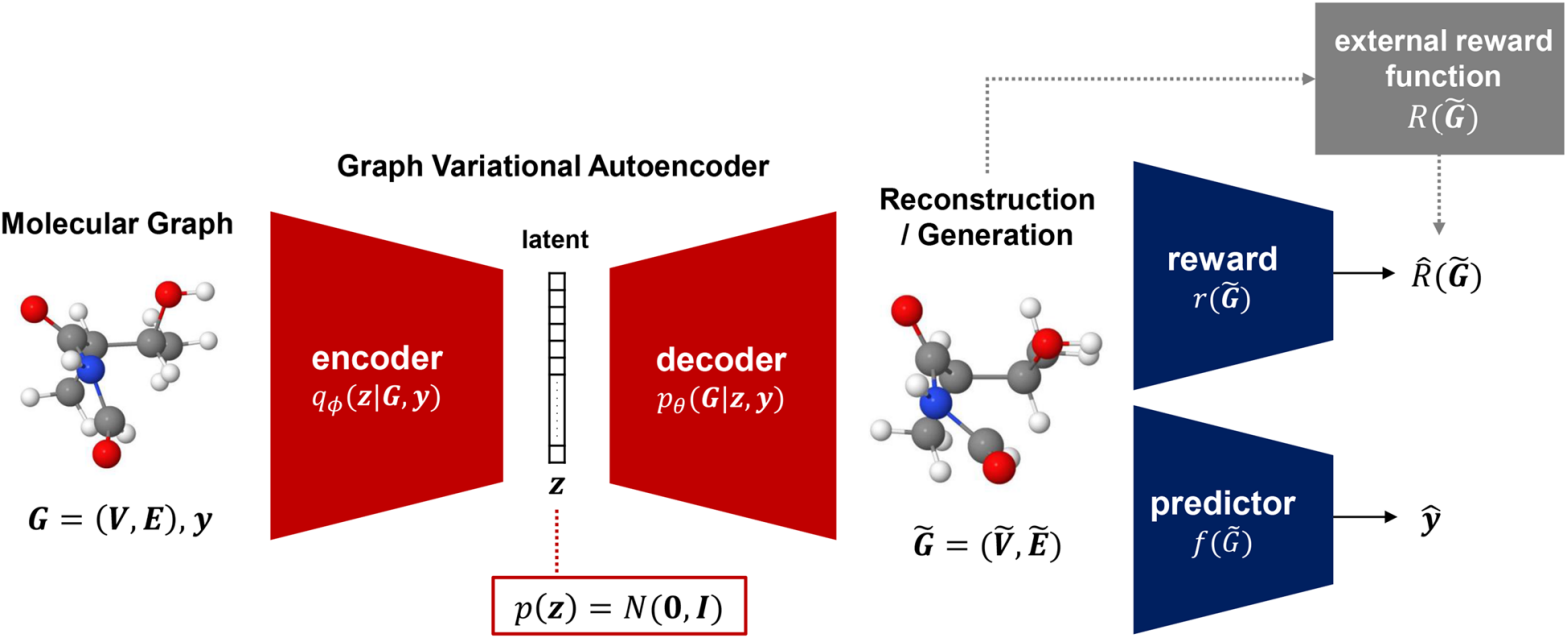
Schematic diagram of model architecture

The architecture of the model is illustrated in Fig. 3. The model consists of five components: the encoder network $q_\phi (\mathbf {z}|\mathcal {G},\mathbf {y})$, decoder network $p_\theta (\mathcal {G}|\mathbf {z},\mathbf {y})$, reward network $r(\mathcal {G})$, predictor network $f(\mathcal {G})$, and external reward function $R(\mathcal {G})$. The encoder network $q_\phi (\mathbf {z}|\mathcal {G},\mathbf {y})$, which corresponds to the approximate posterior distribution, is modeled as message passing neural networks (MPNNs) [13] to be invariant to graph isomorphism. The encoder network takes $\mathcal {G}$ and $\mathbf {y}$ as inputs to produce $\varvec{\mu }_\mathbf {z}(\mathcal {G},\mathbf {y})$ and $\varvec{\sigma }^2_\mathbf {z}(\mathcal {G},\mathbf {y})$, so that $\mathbf {z}$ is sampled from $\mathcal {N}(\mathbf {z}|{\varvec{\mu }_\mathbf {z}(\mathcal {G},\mathbf {y}), \text {diag}(\varvec{\sigma }^2_\mathbf {z}(\mathcal {G},\mathbf {y})}))$ based on the reparameterization trick. The decoder network $p_\theta (\mathcal {G}|\mathbf {z},\mathbf {y})$, which captures the generative distribution, is modeled as a fully-connected neural network. The decoder network takes $\mathbf {z}$ and $\mathbf {y}$ to generate a probabilistic graph $\widetilde{\mathcal {G}}$. The reward and predictor networks are modeled as MPNNs. The reward network $r(\mathcal {G})$ takes $\mathcal {G}$ or $\widetilde{\mathcal {G}}$ as input to predict the reward $R(\mathcal {G})$ or $R(\widetilde{\mathcal {G}})$. The predictor network takes the same input to predict $\mathbf {y}$. The external reward function $R(\mathcal {G})$ is designed based on chemical rules to return a reward of 1 if its input can be decoded as a chemically valid molecular graph and 0 otherwise.

Given *N* molecules and their properties, we form a training dataset $\mathcal {D}=\{\mathcal {G}_t',\mathbf {y}_t\}_{t=1}^N$ with the compressed representation, where $\mathcal {G}_t'=\Phi {(\mathcal {G}_t)}$. Then, the model is trained using the dataset. The objective function for thie training involves the original learning objective of the VAE as well as approximate graph matching, reinforcement learning, and auxiliary property prediction. The details of the model are described in [12].

The training involves the processing of a graph $\mathcal {G}$ in the form of a pair $(\mathbf {V}, \mathbf {E})$ comprising a node matrix $\mathbf {V} \in \mathbb {R}^{|\mathcal {V}| \times p}$, where $\mathbf {V}_i \in \mathbb {R}^{p}$ is the node vector $\mathbf {v}^i \in \mathcal {V}$, and an edge tensor $\mathbf {E}\in \mathbb {R}^{|\mathcal {V}| \times |\mathcal {V}| \times q}$, where $\mathbf {E}_{i,j}\in \mathbb {R}^{q}$ is the edge vector $\mathbf {e}^{i,j} \in \mathcal {E}$ if it corresponds to a bond or substructure and is a zero vector otherwise. This leads to the computational complexity of $O(|\mathcal {V}|^2)$. Because the use of the compressed graph representation directly reduces $|\mathcal {V}|$, the model becomes more scalable to large molecules.

### Molecular graph generation

After training the model, the decoder part $p_\theta (\mathcal {G}|\mathbf {z},\mathbf {y})$ is used to generate new molecular graphs. To generate a molecular graph, we sample $\mathbf {z}_*$ and $\mathbf {y}_*$ from their prior distributions $p(\mathbf {z})$ and $p(\mathbf {y})$. They are fed into the decoder to produce a probabilistic output, which is then decoded via node-wise and edge-wise argmax to obtain a compressed graph $\mathcal {G}'_*$ as

2 $$\begin{aligned} \mathcal {G}'_* =\underset{\mathcal {G}}{\text {argmax }}p_\theta (\mathcal {G}|\mathbf {z}=\mathbf {z}_*,\mathbf {y}=\mathbf {y}_*). \end{aligned}$$

Because $\mathcal {G}'_*$ is originally in the form of the compressed representation, we decompress it into its original representation with the decompression function $\Phi ^{-1}$ as

3 $$\begin{aligned} \mathcal {G}_* = \Phi ^{-1}(\mathcal {G}'_*). \end{aligned}$$

The output $\mathcal {G}_*$ can be interpreted as the chemical structure of a molecule.

## Results and discussion

### GuacaMol benchmark

We investigated the effectiveness of the proposed method using the GuacaMol distribution-learning benchmark [14]. The training dataset for the benchmark is a standardized subset of the ChEMBL database [6], consisting of 1,591,378 molecules with up to 88 heavy atoms.

In the benchmark, the performance of a model for generating chemically valid and diverse molecular graphs is evaluated in terms of *Validity*, *Uniqueness*, and *Novelty* of 10,000 molecular graphs generated by the model. *Validity* is the ratio of valid molecular graphs, for which a molecular graph is counted as valid if it can be processed successfully with RDKit. *Uniqueness* is the ratio of valid graphs that are not duplicates. *Novelty* is the ratio of valid graphs that are not present in the training dataset. In addition, *Kullback-Leibler Divergence (KLD)* and *Fréchet ChemNet Distance (FCD)* are used to evaluate the success of a model in reproducing the distribution of the training dataset.

### Implementation

We used a NAGVAE [12] trained with the training dataset on the compressed graph representation (NAGVAE$_\text {compress}$) as the proposed model. The node and edge features that we used for the compressed representation are listed in Tables 1 and 2, respectively. It should be noted that the type and dimensionality of each feature depend on the training dataset. The model was trained for 10 epochs with a batch size of 10. The hyperparameters in the objective function were set to $\beta _1$=5 and $\beta _2$=1. Other settings were set according to the defaults in [12].

**Table 1:** Node features of compressed graph representation

Feature	Type	Dimensionality
Atom type	One-hot (B, C, N, O, F, Si, P, S, Cl, Se, Br, I)	12
Formal charge	One-hot (-1, 1, 2, 3)	4
No. explicit hydrogens	One-hot (1, 2, 3)	3
Total (*p*)		19

**Table 2:** Edge features of compressed graph representation

Feature	Type	Dimensionality
Bond type	One-hot (single, double, triple, or none)	3
Pattern 1 count	One-hot (1, 2, 3, or none)	3
Pattern 2 count	One-hot (1, 2, 3, or none)	3
Pattern 3 count	One-hot (1, 2, or none)	2
Pattern 4 count	One-hot (1, or none)	1
Pattern 5 count	One-hot (1, 2, or none)	2
Pattern 6 count	One-hot (1, or none)	1
Total (*q*)		15

We employed four SMILES generation models (LSTM [15], VAE [16], AAE [17], and ORGAN [18]) and one molecular graph generation model (GraphMCTS [19]), as implemented in [14], as the baseline models for comparison. SMILES generation models are known to be more scalable to large molecules. The authors of [14] reported that training JTVAE [11] using the GuacaMol benchmark led to an error. We also failed to train the NAGVAE on the original graph representation (NAGVAE$_\text {original}$) [12] owing to an out-of-memory error.

### Molecular graph compression

**Fig. 4:**
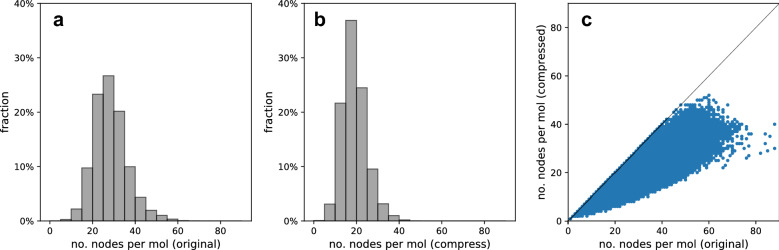
Molecular graph compression results on training dataset: **a** histogram of the number of nodes with the original representation; **b** histogram of the number of nodes with the compressed representation; **c** scatterplot between original and compressed representations

Each molecular graph in the training dataset was compressed using the compressed graph representation. Figure 4 shows the results of molecular graph compression on the dataset, the summary statistics of which are listed in Table 3. The number of nodes with the compressed representation was reduced significantly. By frequency analysis on the dataset, we found that patterns 1–6 appeared 1.10, 1.31, 1.44, 1.03, 0.65, and 0.60 times, respectively, per molecule on average. Subsequently, the average and maximum number of nodes per molecule were reduced by 33.70% and 40.91%, respectively. In the cases of the two largest molecular graphs containing 88 nodes, the numbers of nodes were reduced to 30 and 40 nodes.

**Table 3:** Summary of molecular graph compression results

Statistic	Original rep.	Compressed rep.	Reduction rate (%)
Avg. no. nodes	27.89	18.49	33.70
Max. no. nodes	88	52	40.91

As evident from the results, the compression function $\Phi$ effectively reduced the number of nodes in the molecular graphs. In particular, molecular graphs tended to be better compressed when the number of nodes was large. The high compression rate contributes to reducing the computational cost and memory usage involved in molecular graph generation.

### Molecular graph generation

Table 4 shows a performance comparison between the baseline and proposed models. The experimental results for the baseline models were obtained from [14]. Among the baseline models, GraphMCTS was superior in generating chemically valid and diverse molecular graphs in terms of the validity, uniqueness, and novelty scores. LSTM yielded better performance in reproducing the underlying property distributions of the training dataset in terms of the KLD and FCD scores. JTVAE and NAGVAE$_\text {original}$ failed to provide results owing to the scalability issue. The proposed model, NAGVAE$_\text {compress}$, was successful in generating molecular graphs. Notably, NAGVAE$_\text {compress}$ yielded comparable or superior performance in terms of the validity, uniqueness, and novelty scores. One drawback was the low distribution learning performance. It yielded lower KLD and FCD scores compared to the SMILES generation models.

**Table 4:** Molecular graph generation results of baseline and proposed models

Metric	SMILES-based				Graph-based			
	LSTM	VAE	AAE	ORGAN	GraphMCTS	JTVAE	NAGVAE$_\text {original}$	NAGVAE$_\text {compress}$
Validity	0.959	0.870	0.822	0.379	1.000			0.927
Uniqueness	1.000	0.999	1.000	0.841	1.000			0.955
Novelty	0.912	0.974	0.998	0.687	0.994	N/A	N/A	1.000
KLD	0.991	0.982	0.886	0.267	0.522			0.384
FCD	0.913	0.863	0.529	0.000	0.015			0.009

From a computational perspective, the use of the compressed representation reduced the computational burden for both the training and inference phases. Considering the complexity $\mathcal {O}(|\mathcal {V}|^2)$ which increases with the number of nodes, training and inference on a more compact representation with a smaller number of nodes are faster and require lower computational cost and memory usage. This is also evident from the fact that NAGVAE$_\text {original}$ failed to be trained, whereas NAGVAE$_\text {compress}$ was successfully trained with the training dataset. Additionally, the decompression for the compressed graph representation had little effect on the computational burden. The molecular graph generation by NAGVAE$_\text {compress}$, which involves inference with the decoder network $p_\theta (\mathcal {G}|\mathbf {z},\mathbf {y})$ and decompression with the function $\Phi ^{-1}$, only took around 0.004 s and 0.001 s per molecular graph on average for the inference and decompression, respectively.

As demonstrated by the experimental results, the use of compressed graph representation makes molecular graph generation scalable to large molecular graphs without performance degradation with regard to the generation of chemically valid and diverse molecular graphs. We expect that molecular graph compression will shed some light on improving the efficiency and scalability of other molecular graph generation methods without sacrificing their performance.

## Conclusion

In this paper, we presented a molecular graph compression method to address the scalability issue of molecular graph generation. We identified six small substructural patterns that commonly appear between atom pairs in real-world molecules. Given a molecular graph, we converted the relevant substructures into new edges by representing them using additional edge features in the compressed graph representation. A generative model was constructed in a more efficient and scalable manner by training the model on the compressed representation. By conducting an experimental investigation using the GuacaMol benchmark, we found that the proposed method reduced the number of nodes significantly without any information loss. The generative model constructed on the compressed representation achieved performance comparable to that of the baseline methods regarding molecular graph generation.

Although mitigating the high computational complexity intrinsically imposed on molecular graph generation has been challenging, this work successfully demonstrated that the molecular graph compression approach can effectively alleviate the complexity. We expect that this approach will be more effective with the better identification of data-specific substructural patterns that can be regarded as edge features. The use of the compressed representation contributes to a substantial reduction in the computational cost and memory usage, making it scalable to large molecules. This approach can be applied to other molecular graph generation methods to improve their efficiency and scalability, which merits further investigations.

## Data Availability

The source code used in this study is available online at http://github.com/seokhokang/graphvae_compress/. The source code and dataset for GuacaMol benchmark are publicly accessible from https://github.com/BenevolentAI/guacamol/.
